# Supplementary figures and images for: Polylactic acid as a suitable material for 3D printing of protective masks in times of COVID-19 pandemic
Source: PeerJ. 2020 Oct 29;8:e10259. doi: 10.7717/peerj.10259 (PMC7603793; doi:10.7717/peerj.10259)

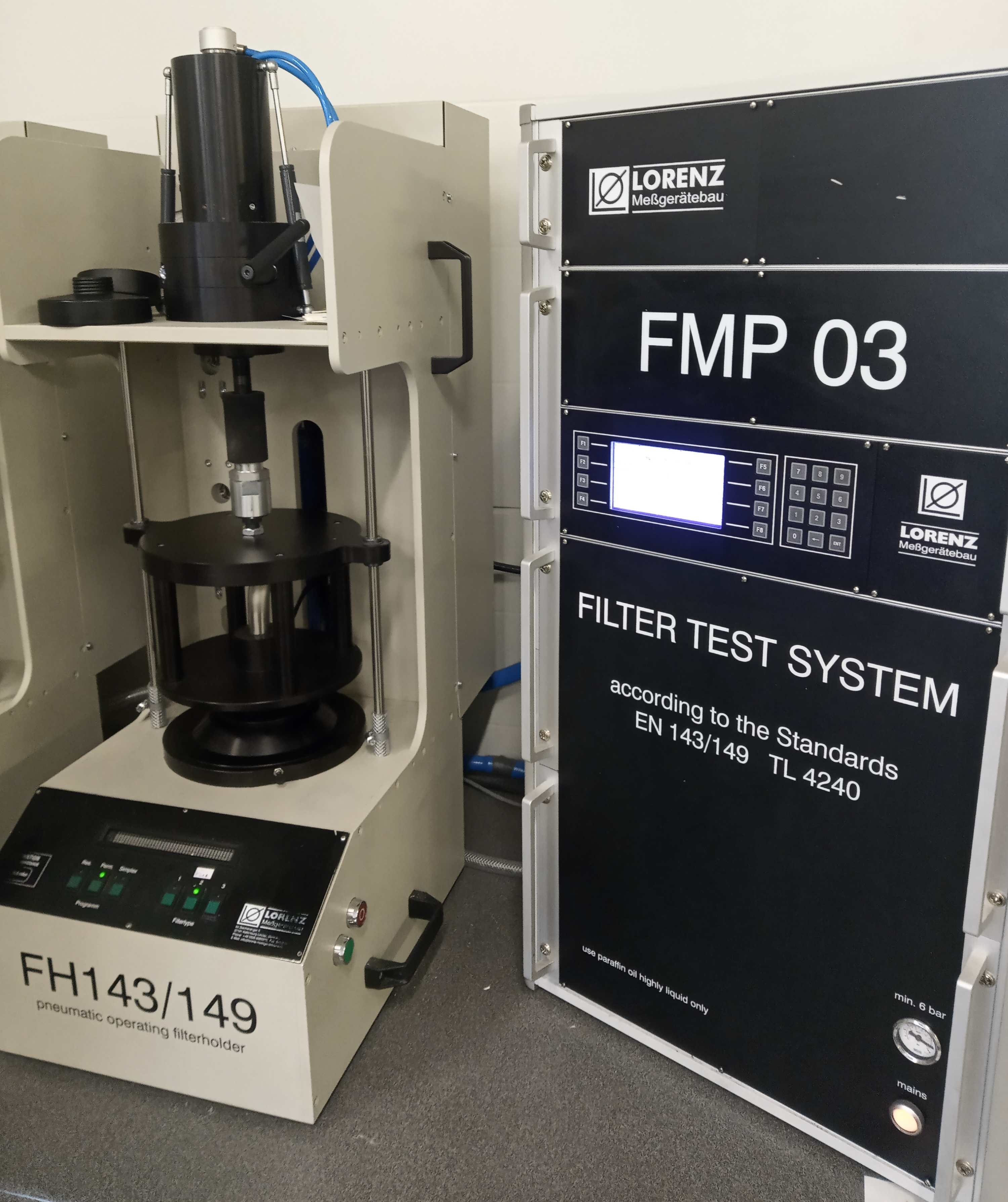

Supplement: Supplemental Information 1 — The device consists of two parts, main control unit with aerosol generator and laser photometer (on the right of the image) and pneumatically operated filter-holder (on the left of the image). [file peerj-08-10259-s001.png]

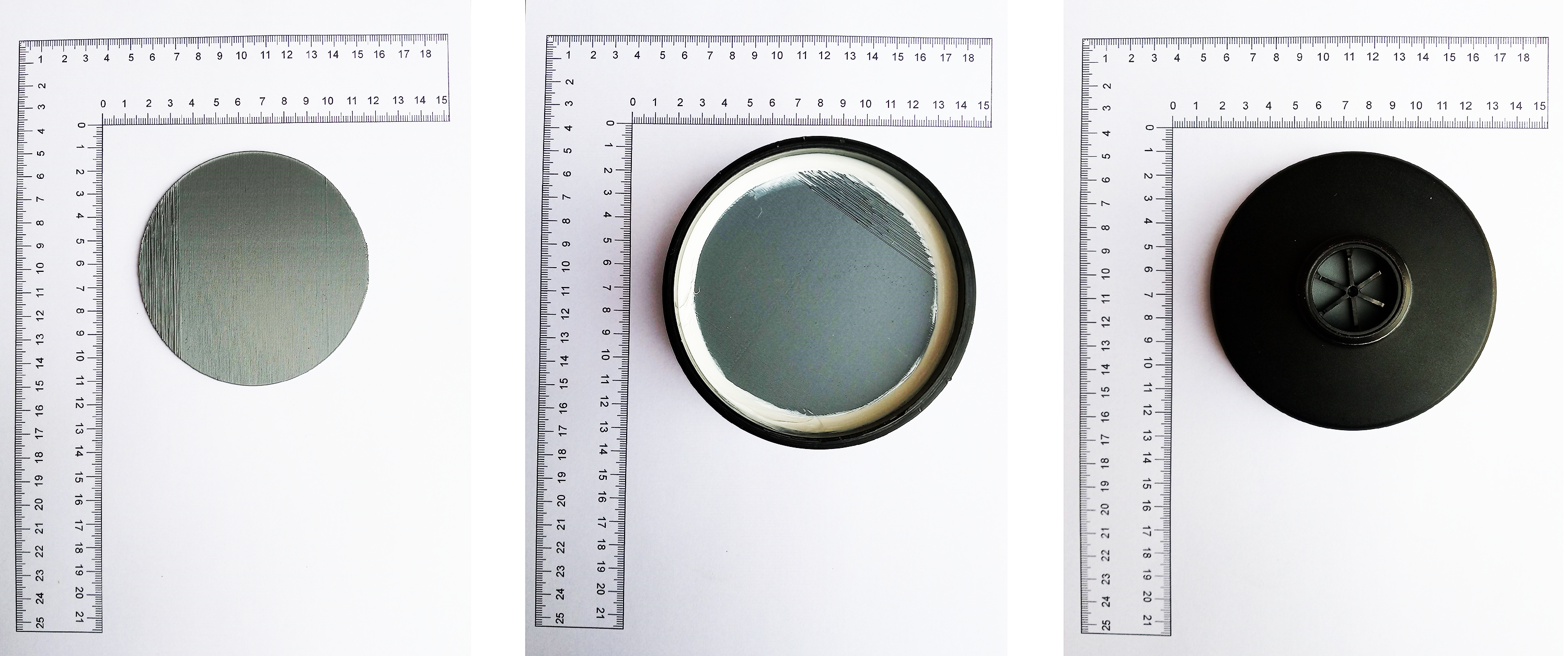

Supplement: Supplemental Information 2 — On the left of the image is a printed PLA sample. The middle part presents PLA sample attached into standardized Px filter cartridge with a sealer. On the right of the image is presented the whole setup with standardized Px filter cartridge, ready for testing. [file peerj-08-10259-s002.png]

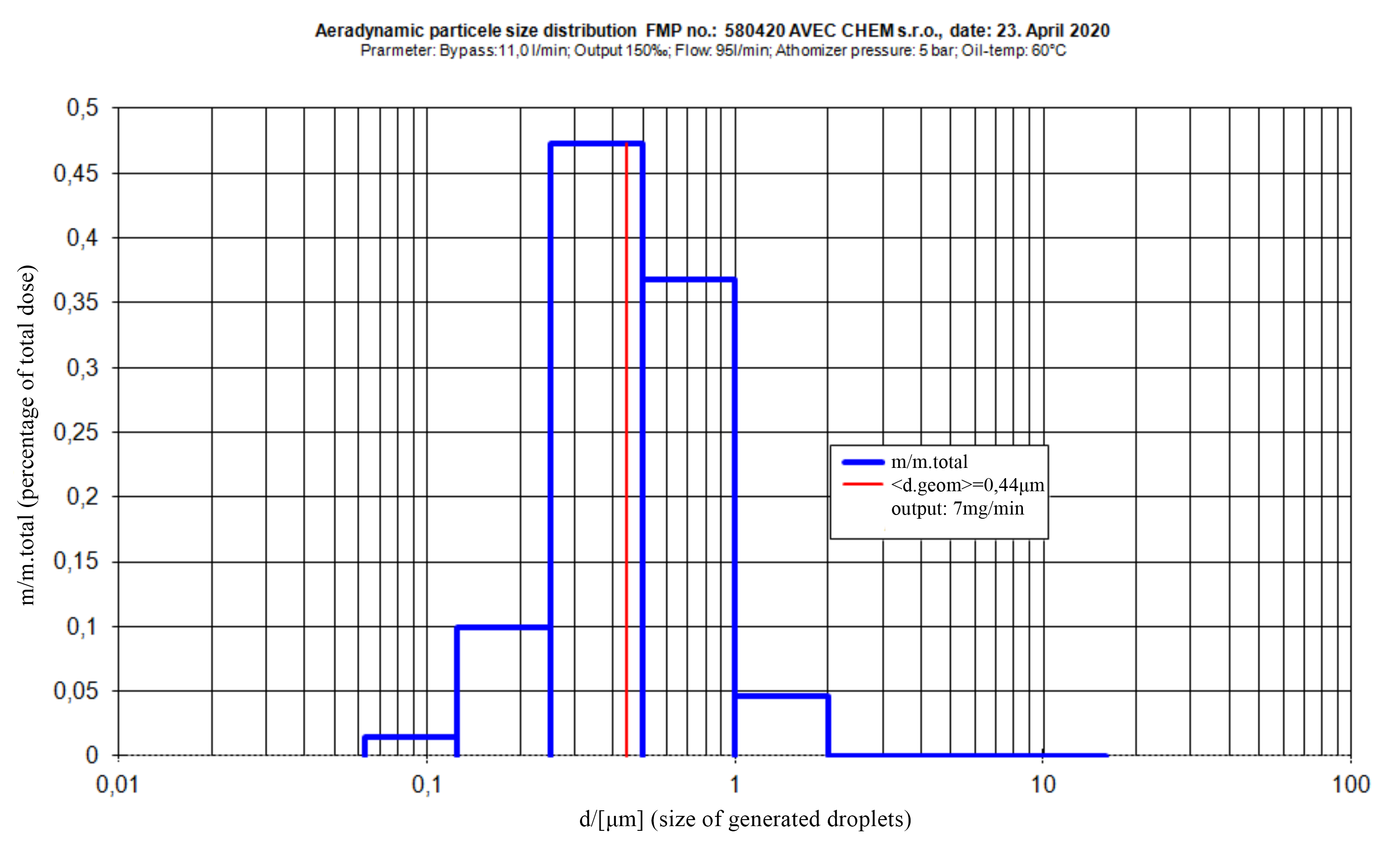

Supplement: Supplemental Information 3 — On the x-axis is presented size of generated droplets (μm), on the y-axis is presented a percentage of the total dose. Red line represents geomean of particles, blue line represents a distribution of particles, defined by size and percentage. [file peerj-08-10259-s003.png]

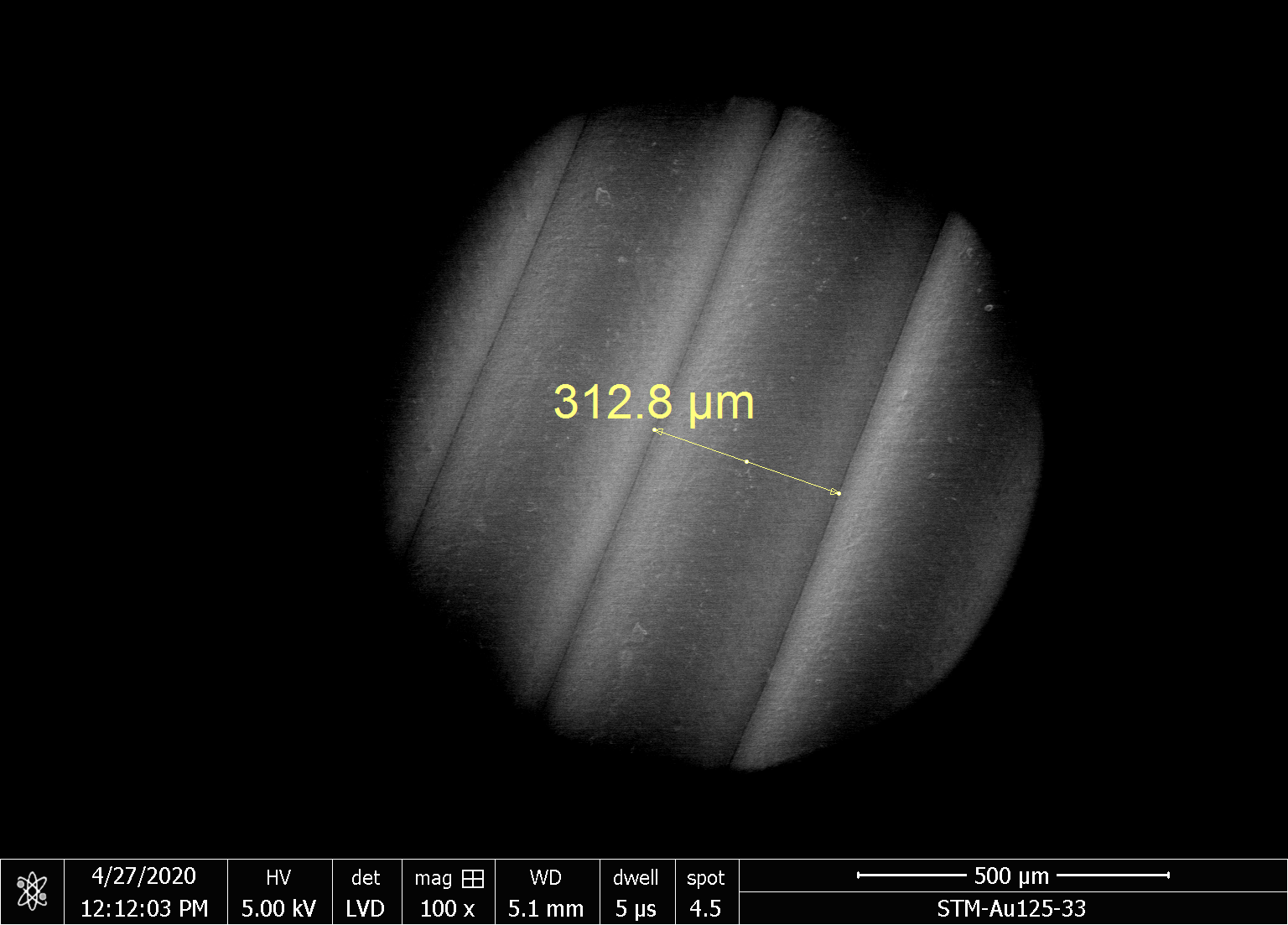

Supplement: Supplemental Information 4 — SEM parameters: low vacuum, 5 kV, LVD detector, dwell time 5 µs, spot size 4.5. Image was taken at 100× magnification (scale bar = 500 µm). The observed gap was measured and marked by yellow line. [file peerj-08-10259-s004.png]

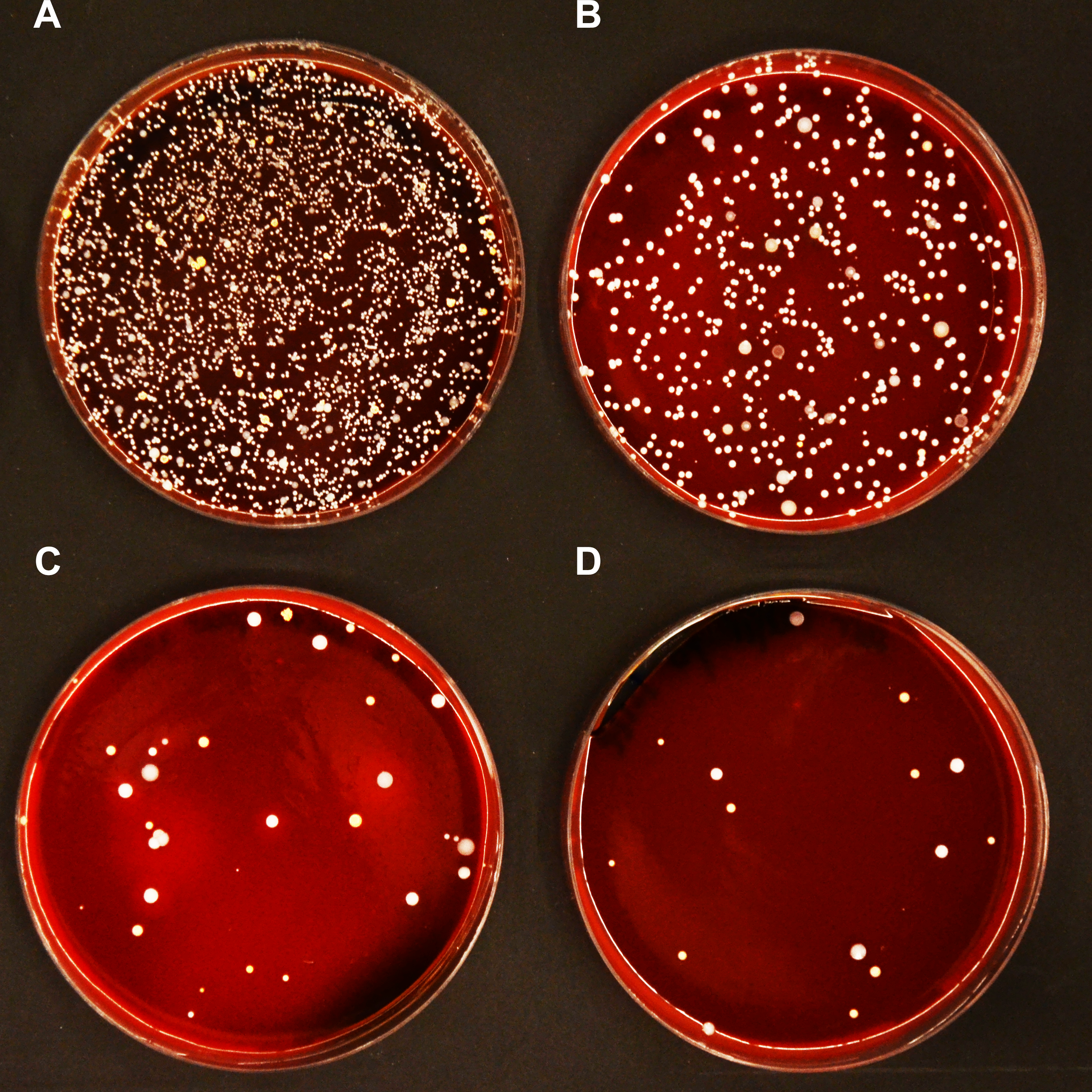

Supplement: Supplemental Information 5 — A representative set of blood agar plates is displayed. The plates were inoculated with material collected from (A) inner surface of the mask before treatment; (B) inner surface of the mask disinfected by short rinsing by ethanol; (C) outer surface of the mask before treatment; (D) outer surface of the mask disinfected by short rinsing by ethanol. [file peerj-08-10259-s005.png]
