# Supplementary material for: Polylactic acid as a suitable material for 3D printing of protective masks in times of COVID-19 pandemic
Source: PeerJ. 2020 Oct 29;8:e10259. doi: 10.7717/peerj.10259 (PMC7603793; doi:10.7717/peerj.10259)
Supplement: Supplemental Information 6 — Infectivity of control SARS-CoV-2 virus. [file peerj-08-10259-s006.docx]

|  | Final conc. CTE  (%) (%) | | |  | Final conc. CTE  (%) (%) | | | | Final conc. CTE Dilution  (%) (%) | | | | |  |  | | Infectivity  (%) | |  |  |
| --- | --- | --- | --- | --- | --- | --- | --- | --- | --- | --- | --- | --- | --- | --- | --- | --- | --- | --- | --- | --- |
| ethanol | 9.6 | 100 | isopropanol | | | 7.0 | 100 | sodium  hypochlorite | | 0.085 | 100 | Control-SARS-CoV-2 | 0 | | | 100 | |  |  |  |
|  | 3.84 | 100 |  |  |  | 2.8 | 0 |  |  | 0.034 | 100 |  | 1/2.5 | | | 100 | |  |  |  |
|  | 1.536 | 100 |  |  |  | 1.12 | 0 |  |  | 0.014 | 0 |  | 1/6.3 | | | 100 | |  |  |  |
|  | 0.614 | 100 |  |  |  | 0.448 | 0 |  |  | 0.005 | 0 |  | 1/15.6 | | | 100 | |  |  |  |
|  | 0.246 | 100 |  |  |  | 0.179 | 0 |  |  | 0.002 | 0 |  | 1/39.1 | | | 100 | |  |  |  |
|  | 0.098 | 0 |  |  |  | 0.072 | 0 |  |  | 0.0009 | 0 |  | 1/97.7 | | | 33.3 | |  |  |  |
|  | 0.039 | 0 |  |  |  | 0.029 | 0 |  |  | 0.00035 | 0 |  | 1/244.1 | | | 33.3 | |  |  |  |
|  | 0.016 | 0 |  |  |  | 0.011 | 0 |  |  | 0.00014 | 0 |  | 1/1525.9 | | | 0 | |  |  |  |
