# Supplementary material for: Polylactic acid as a suitable material for 3D printing of protective masks in times of COVID-19 pandemic
Source: PeerJ. 2020 Oct 29;8:e10259. doi: 10.7717/peerj.10259 (PMC7603793; doi:10.7717/peerj.10259)
Supplement: Supplemental Information 7 — Results are expressed in 103 genome copies/mL, representing the mean of triplicate tests. Untreated samples represent genome copies/mL of carrier without treatment by disinfectant. [file peerj-08-10259-s007.docx]

Supplementary Table 1. PLA material contaminated by HAdV, untreated or treated with ethanol, isopropanol or sodium hypochlorite. Results are expressed in 10^3^ genome copies/mL, representing the mean of triplicate tests. Untreated samples represent genome copies/mL of carrier without treatment by disinfectant.

| Virus genome copies recovered from PLA carriers (10^3^ genome copies/mL) | | | | |  |
| --- | --- | --- | --- | --- | --- |
|  | untreated | ethanol | isopropanol | sodium hypochlorite | |
| HAdV | 150 | 86.8 | 41.4 | 0 | |
