# Supplementary material for: Polylactic acid as a suitable material for 3D printing of protective masks in times of COVID-19 pandemic
Source: PeerJ. 2020 Oct 29;8:e10259. doi: 10.7717/peerj.10259 (PMC7603793; doi:10.7717/peerj.10259)
Supplement: Supplemental Information 8 — PLA material contaminated by Staphylococcus epidermidis, Escherichia coli and Candida albicans, untreated or treated with ethanol, isopropanol or sodium hypochlorite. Results are expressed in CFU/mL, as the individual values of triplicate tests. Untreated samples indicate the CFU/mL count present on contaminated carriers. [file peerj-08-10259-s008.docx]

| Colony forming units on PLA material after contamination by *S. epidermidis* (CFU/mL) | | | | |  |
| --- | --- | --- | --- | --- | --- |
|  | untreated | ethanol | isopropanol | sodium hypochlorite |  |
| Experiment 1 | 9×10^4^ | 0 | 0 | 0 |  |
| Experiment 2 | 1×10^5^ | 0 | 2 | 1 |  |
| Experiment 3 | 6×10^4^ | 0 | 0 | 0 |  |

| Colony forming units on PLA material after contamination by *E. coli* (CFU/mL) | | | | |  |
| --- | --- | --- | --- | --- | --- |
|  | untreated | ethanol | isopropanol | sodium hypochlorite |  |
| Experiment 1 | 1×10^5^ | 0 | 0 | 0 |  |
| Experiment 2 | 8×10^4^ | 0 | 0 | 0 |  |
| Experiment 3 | 1.8×10^5^ | 0 | 1 | 0 |  |

| Colony forming units on PLA material after contamination by *C. albicans* (CFU/mL) | | | | |  |
| --- | --- | --- | --- | --- | --- |
|  | untreated | ethanol | isopropanol | sodium hypochlorite |  |
| Experiment 1 | 3.5×10^4^ | 0 | 0 | 0 |  |
| Experiment 2 | 3.3×10^4^ | 0 | 0 | 0 |  |
| Experiment 3 | 4×10^4^ | 0 | 0 | 0 |  |
