# Supplementary material for: Polylactic acid as a suitable material for 3D printing of protective masks in times of COVID-19 pandemic
Source: PeerJ. 2020 Oct 29;8:e10259. doi: 10.7717/peerj.10259 (PMC7603793; doi:10.7717/peerj.10259)

SARS-CoV-2 brightfield

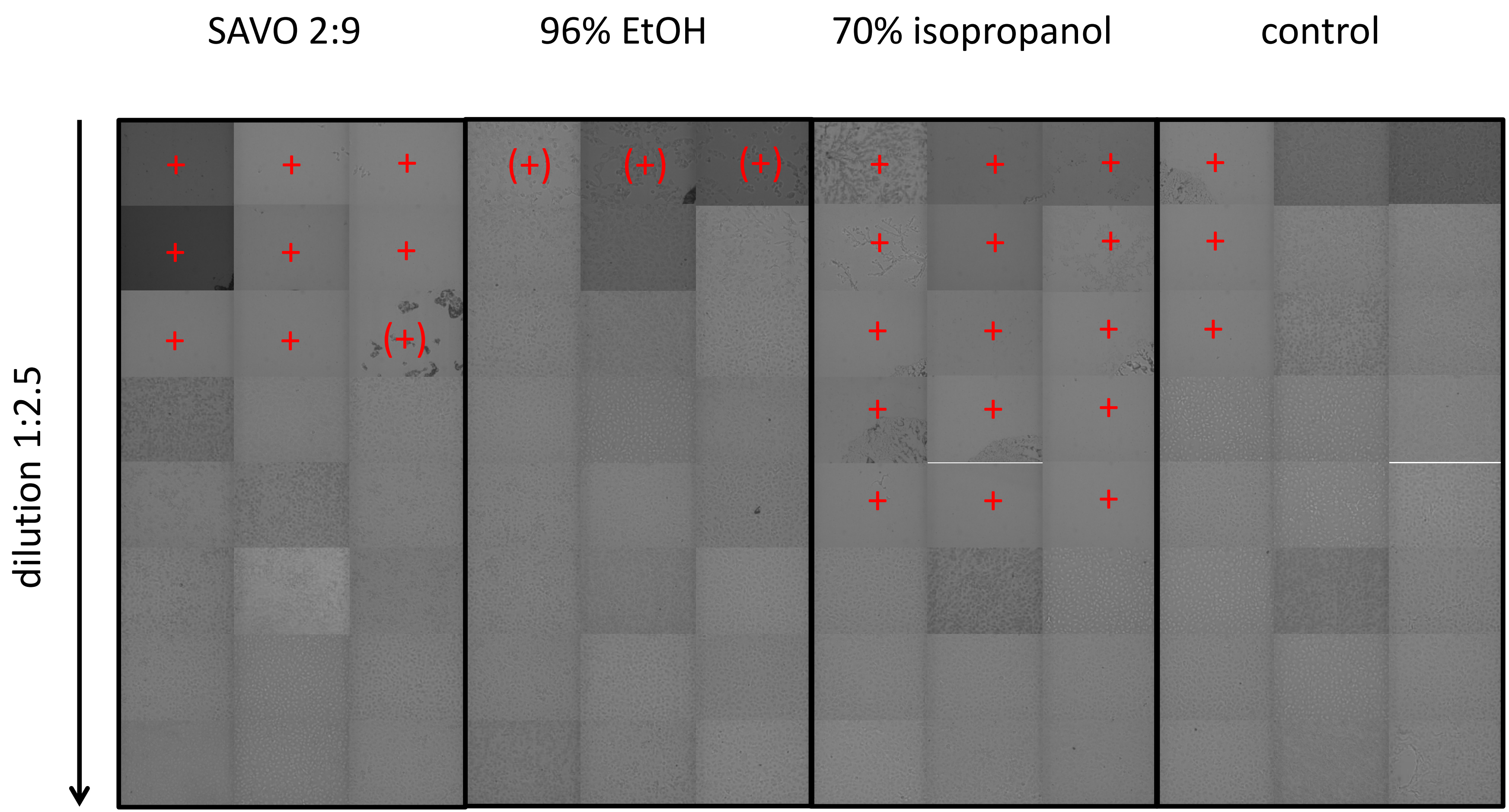

Cell detachment

SARS-CoV-2 Texas Red

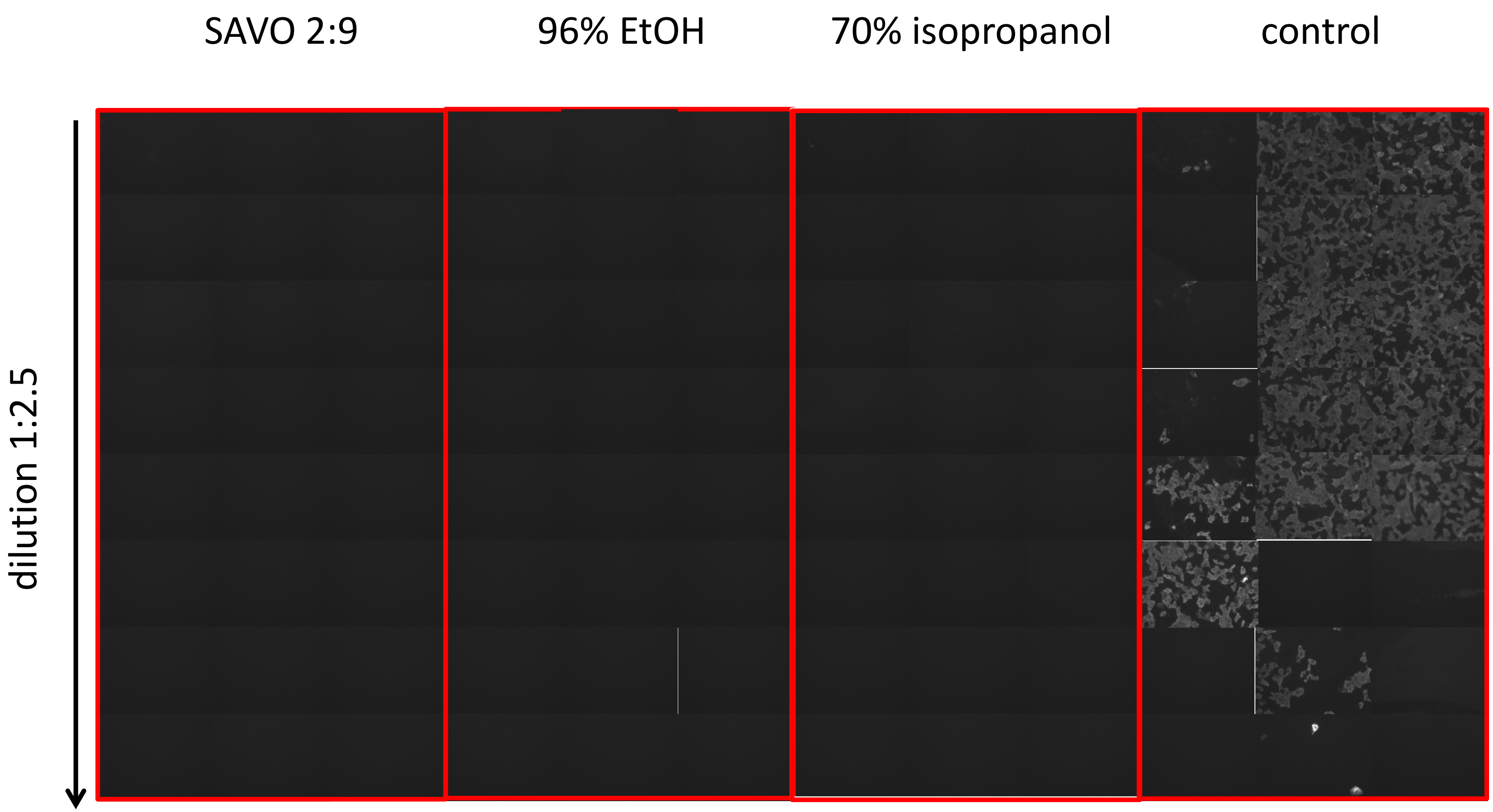

Supplement: Supplemental Information 9 — PLA material contaminated by SARS-CoV-2, untreated or treated with ethanol, isopropanol or sodium hypochlorite. Results are depicted as microimages from brightfield microscopy and immunofluorescence assay. [file peerj-08-10259-s009.pdf]
