# Supplementary material for: Polylactic acid as a suitable material for 3D printing of protective masks in times of COVID-19 pandemic
Source: PeerJ. 2020 Oct 29;8:e10259. doi: 10.7717/peerj.10259 (PMC7603793; doi:10.7717/peerj.10259)
Supplement: Supplemental Information 10 — PLA material contaminated by HAdV, untreated or treated with ethanol, isopropanol or sodium hypochlorite. Results are expressed in 103 IU/mL, as the individual values of triplicate tests. Untreated samples indicate the virus count on contaminated carriers in IU/mL. [file peerj-08-10259-s010.docx]

| HAdV recovered from PLA carriers (10^3^ IU/mL) | | | | |  |
| --- | --- | --- | --- | --- | --- |
|  | untreated | ethanol | isopropanol | sodium hypochlorite | |
| Experiment 1 | 7.5 | 0 | 0 | 0 | |
| Experiment 2 | 5.6 | 0.004 | 2.4 | 0 | |
| Experiment 3 | 1 000 | 4.2 | 4.7 | 0 | |

| HAdV genome copies recovered from PLA carriers (10^3^ genome copies/mL) | | | | |  |
| --- | --- | --- | --- | --- | --- |
|  | untreated | ethanol | isopropanol | sodium hypochlorite | |
| Experiment 1 | 14.7 | 96.5 | 73.4 | 0 | |
| Experiment 2 | 21.4 | 51.8 | 23.4 | 0 | |
| Experiment 3 | 414.0 | 112.0 | 27.4 | 0 | |
