# Supplementary material for: Polylactic acid as a suitable material for 3D printing of protective masks in times of COVID-19 pandemic
Source: PeerJ. 2020 Oct 29;8:e10259. doi: 10.7717/peerj.10259 (PMC7603793; doi:10.7717/peerj.10259)
Supplement: Supplemental Information 11 [file peerj-08-10259-s011.pdf]

control sample

spot 1

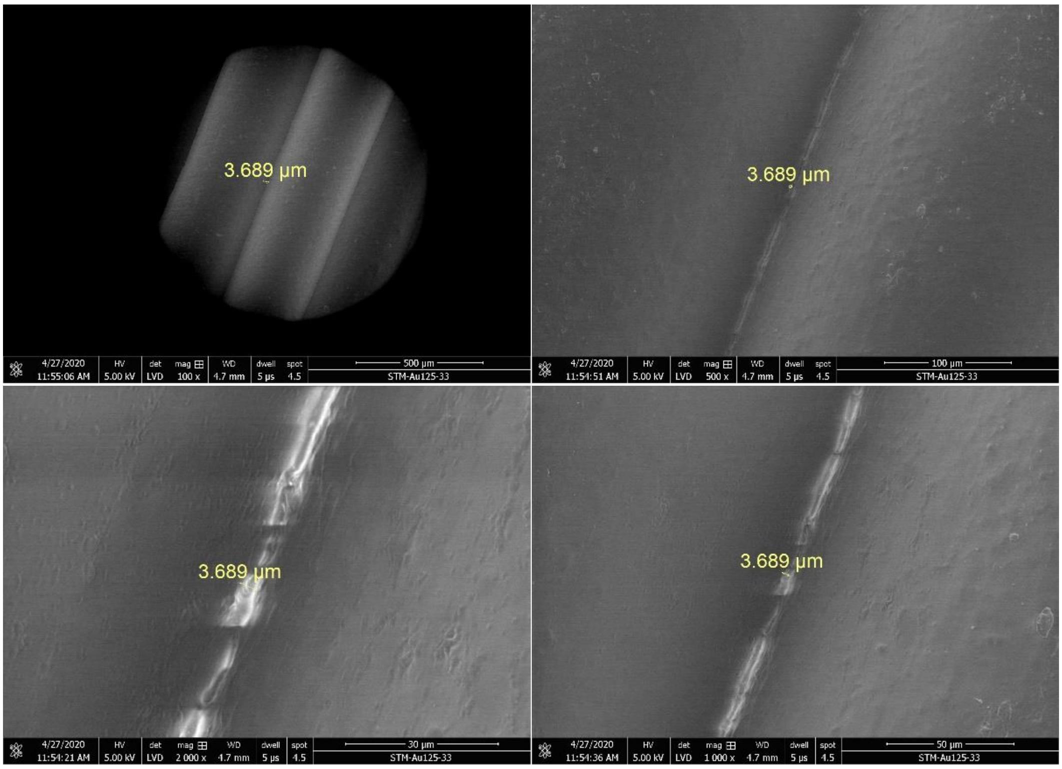

spot 2

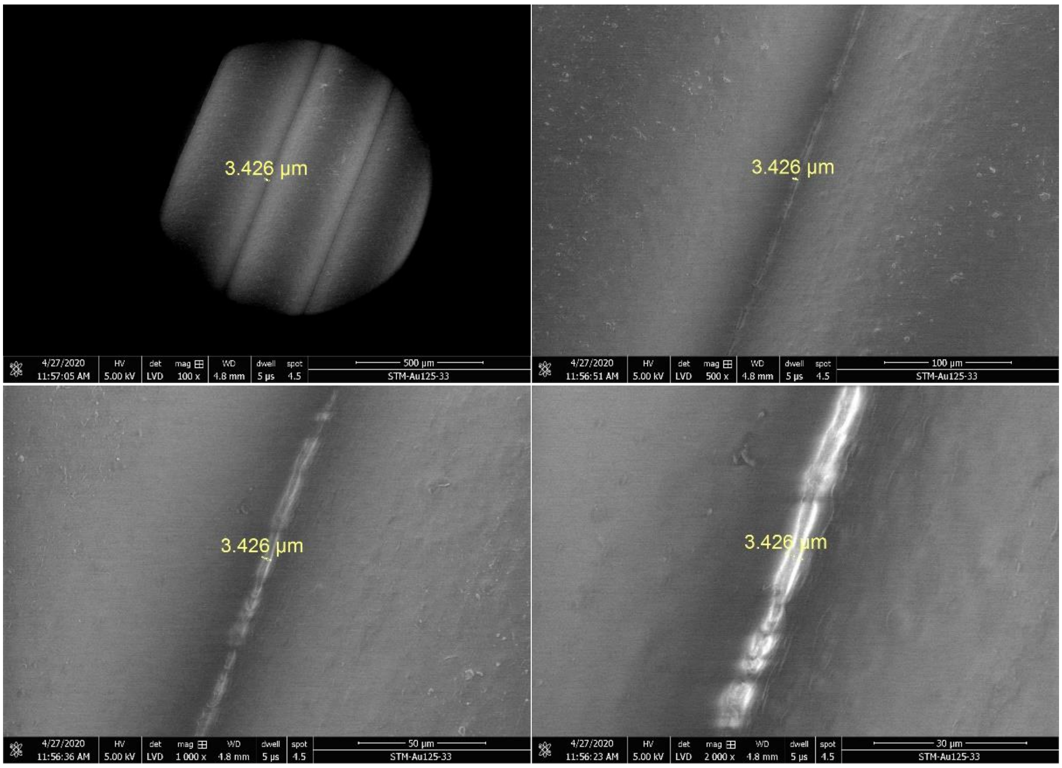

spot 3

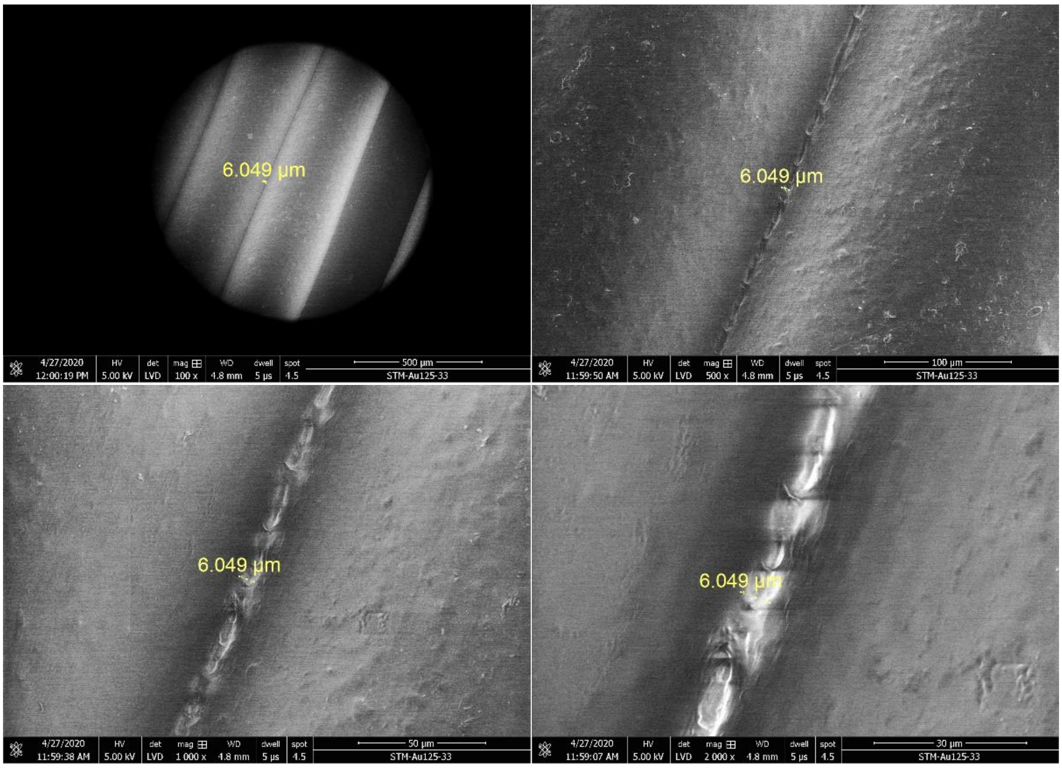

6.049 µm
4/27/2020 12:00:19 PM
HV 5.00 kV
det LVD
mag 100 x
WD 4.8 mm
dwell 5 µs
spot 4.5
500 µm
STM-Au125-33
6.049 µm
4/27/2020 11:59:50 AM
HV 5.00 kV
det LVD
mag 500 x
WD 4.8 mm
dwell 5 µs
spot 4.5
100 µm
STM-Au125-33
6.049 µm
4/27/2020 11:59:38 AM
HV 5.00 kV
det LVD
mag 1 000 x
WD 4.8 mm
dwell 5 µs
spot 4.5
50 µm
STM-Au125-33
6.049 µm
4/27/2020 11:59:07 AM
HV 5.00 kV
det LVD
mag 2 000 x
WD 4.8 mm
dwell 5 µs
spot 4.5
30 µm
STM-Au125-33

ethanol 5×15 min

spot 1

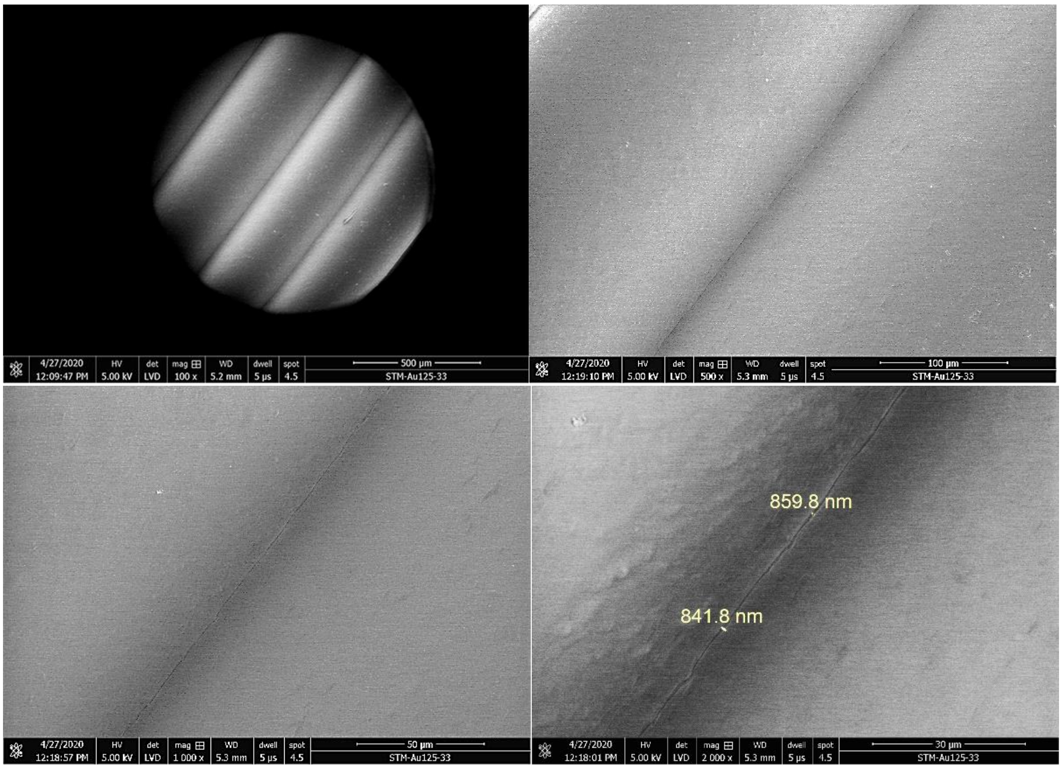

spot 2

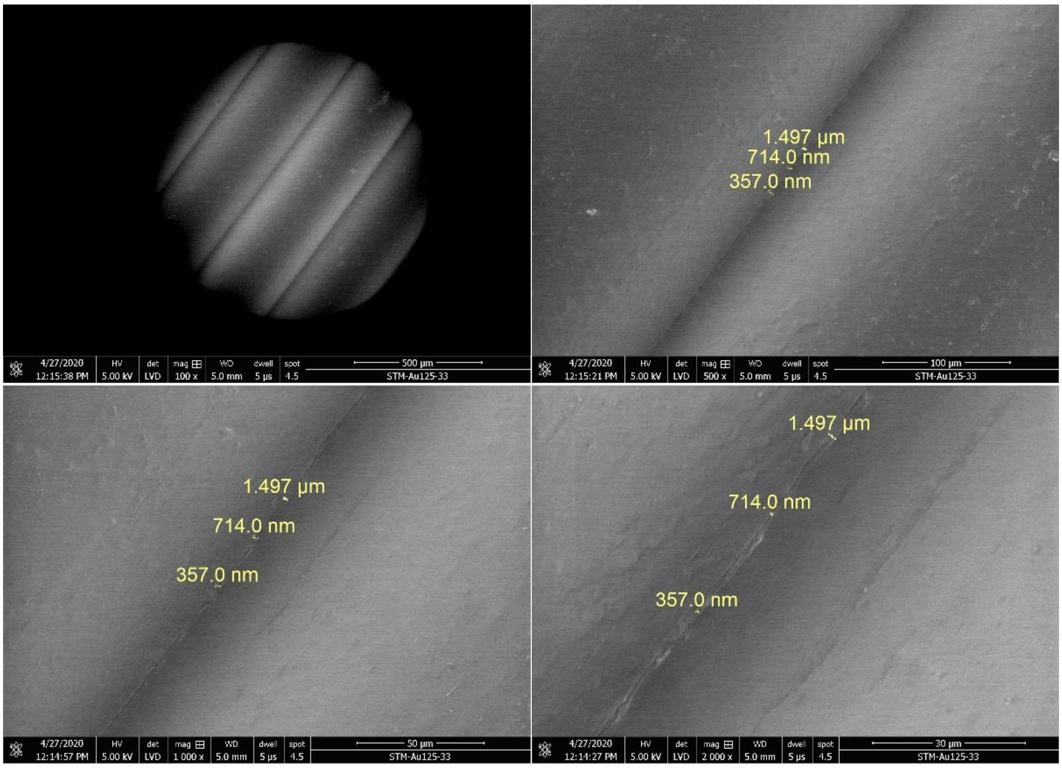

spot 3

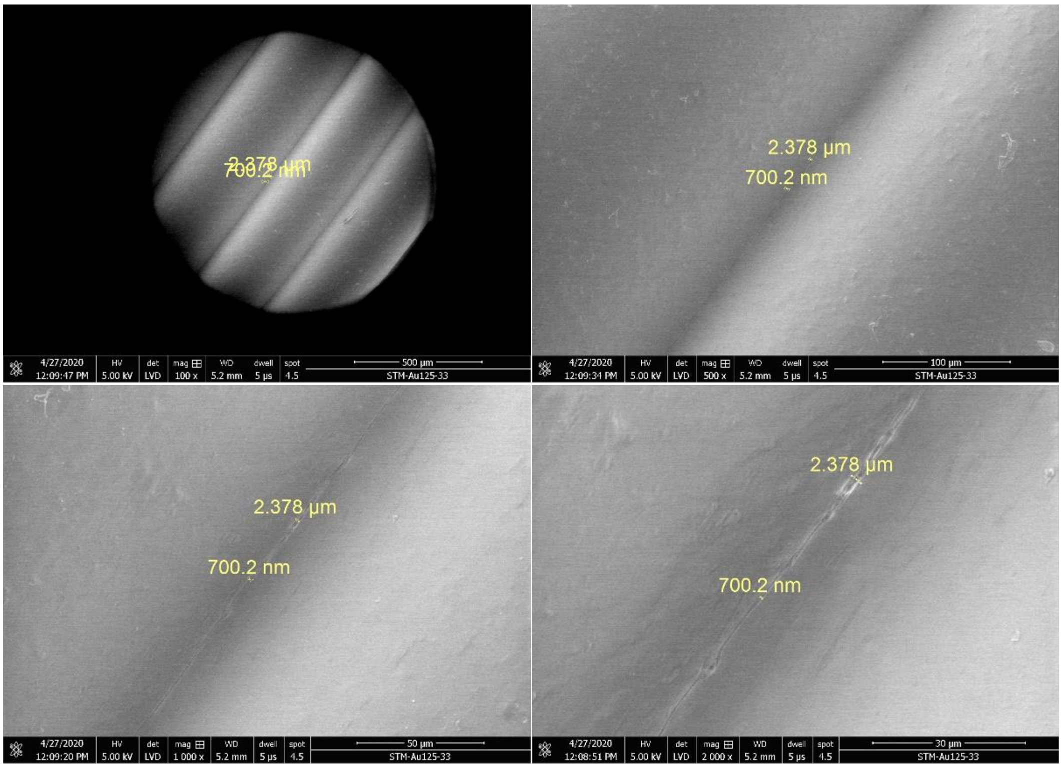
2.378 µm
700.2 nm
4/27/2020 12:09:47 PM | HV 5.00 kV | det LVD | mag 100 x | WD 5.2 mm | dwell 5 µs | spot 4.5 | 500 µm | STM-Au125-33
2.378 µm
700.2 nm
4/27/2020 12:09:34 PM | HV 5.00 kV | det LVD | mag 500 x | WD 5.2 mm | dwell 5 µs | spot 4.5 | 100 µm | STM-Au125-33
2.378 µm
700.2 nm
4/27/2020 12:09:20 PM | HV 5.00 kV | det LVD | mag 1 000 x | WD 5.2 mm | dwell 5 µs | spot 4.5 | 50 µm | STM-Au125-33
2.378 µm
700.2 nm
4/27/2020 12:08:51 PM | HV 5.00 kV | det LVD | mag 2 000 x | WD 5.2 mm | dwell 5 µs | spot 4.5 | 30 µm | STM-Au125-33

isopropanol 5×15 min

spot 1

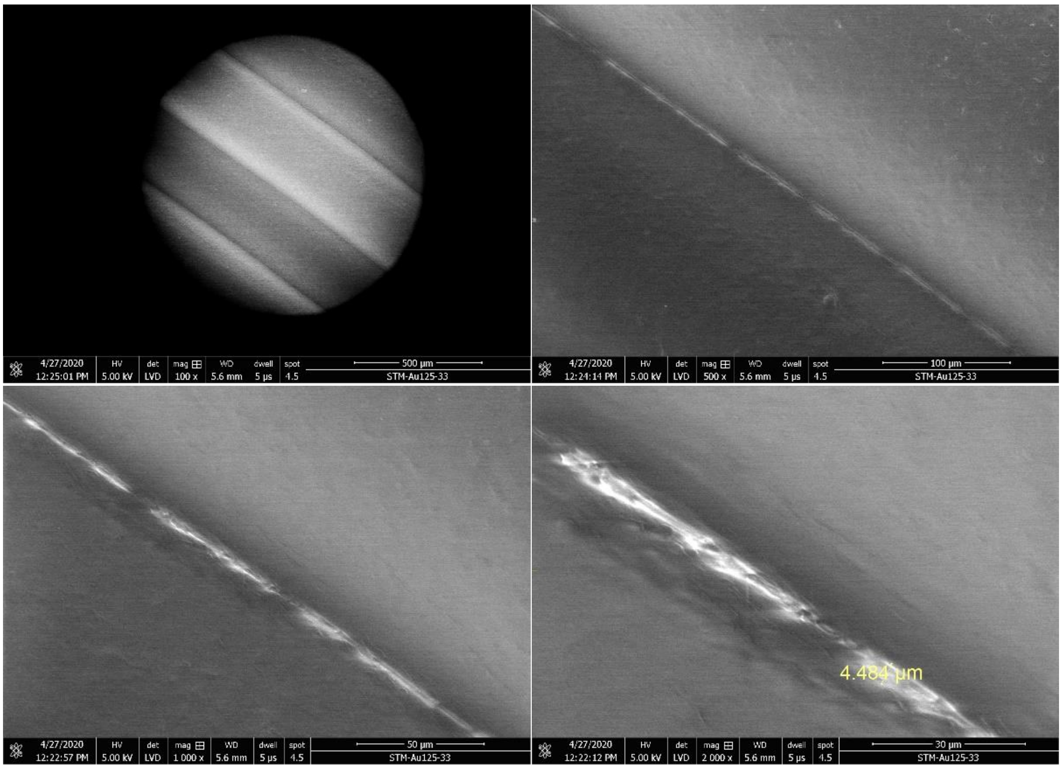

spot 2

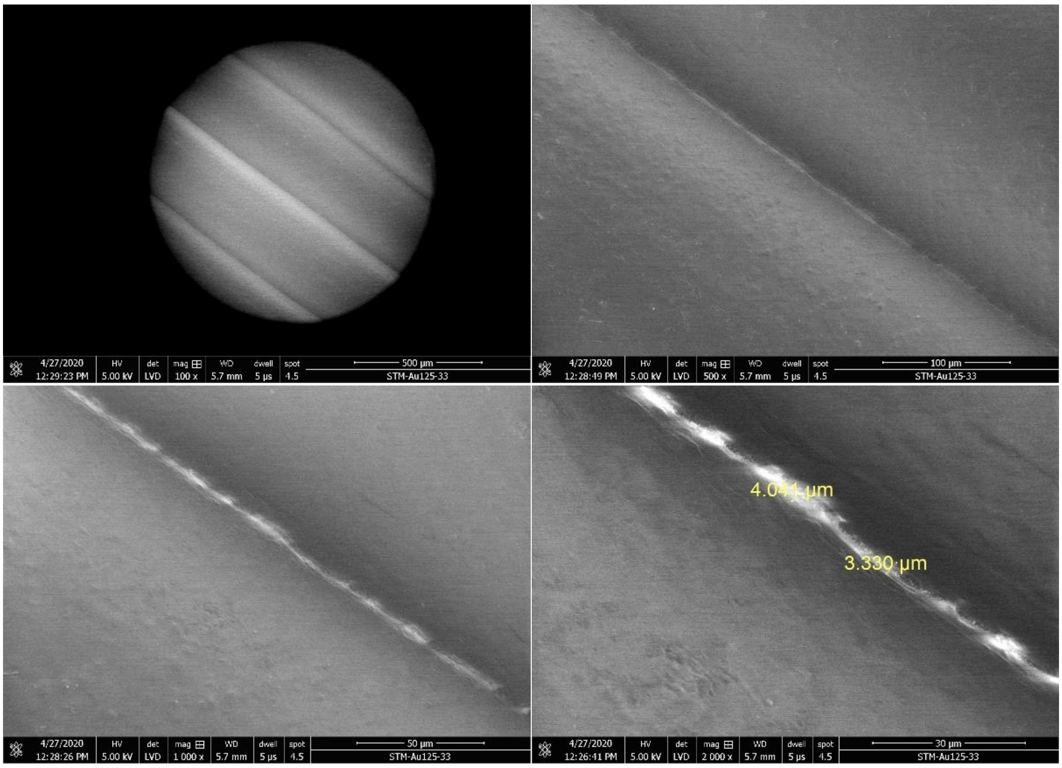

spot 3

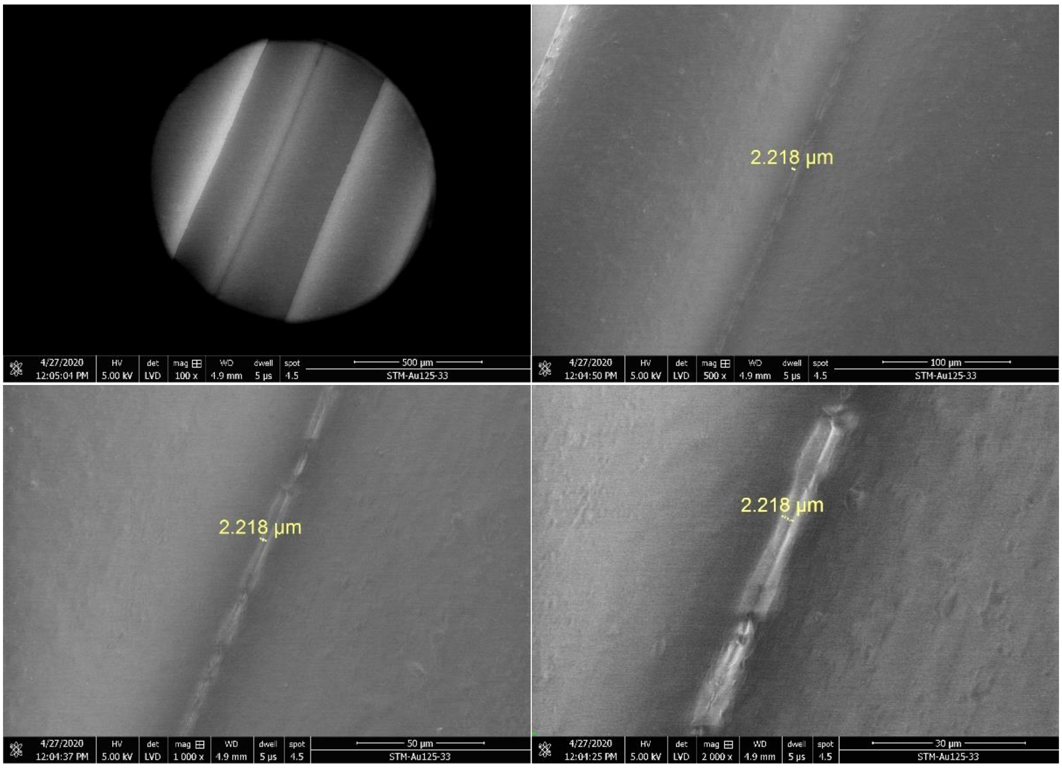

4/27/2020
12:05:04 PM
HV
5.00 kV
det
LVD
mag
100 x
WD
4.9 mm
dwell
5 µs
spot
4.5
500 µm
STM-Au125-33
2.218 µm
4/27/2020
12:04:50 PM
HV
5.00 kV
det
LVD
mag
500 x
WD
4.9 mm
dwell
5 µs
spot
4.5
100 µm
STM-Au125-33
2.218 µm
4/27/2020
12:04:37 PM
HV
5.00 kV
det
LVD
mag
1 000 x
WD
4.9 mm
dwell
5 µs
spot
4.5
50 µm
STM-Au125-33
2.218 µm
4/27/2020
12:04:25 PM
HV
5.00 kV
det
LVD
mag
2 000 x
WD
4.9 mm
dwell
5 µs
spot
4.5
30 µm
STM-Au125-33

sodium hypochlorite 5×15 min

spot 1

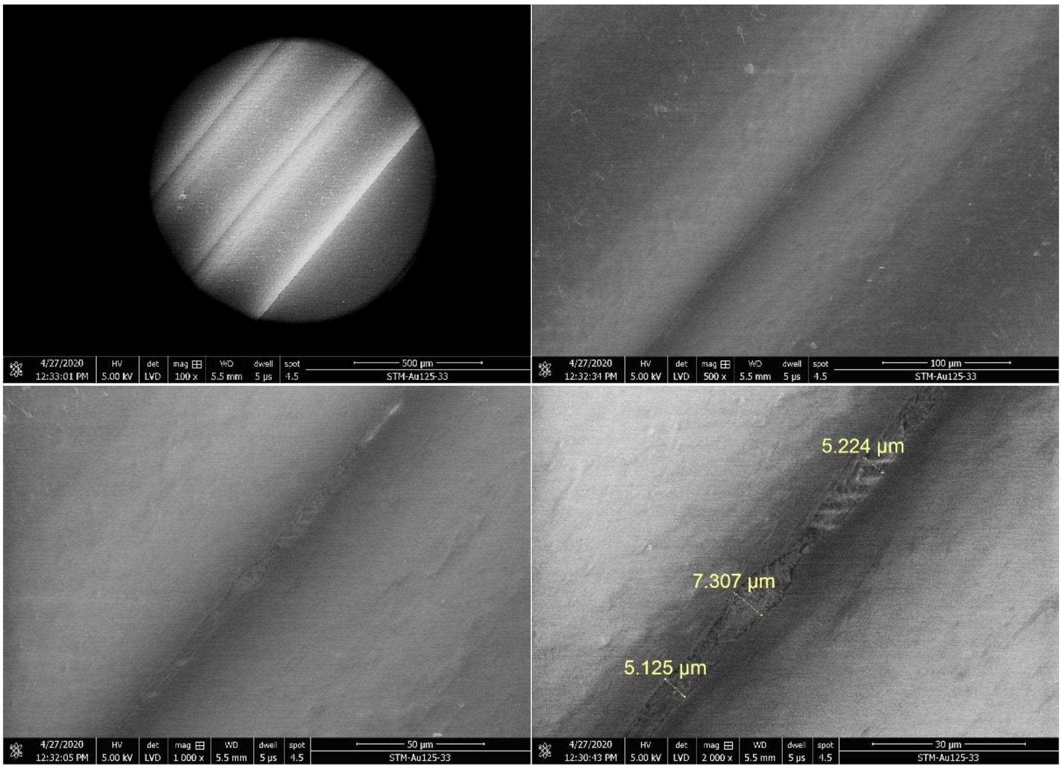

spot 2

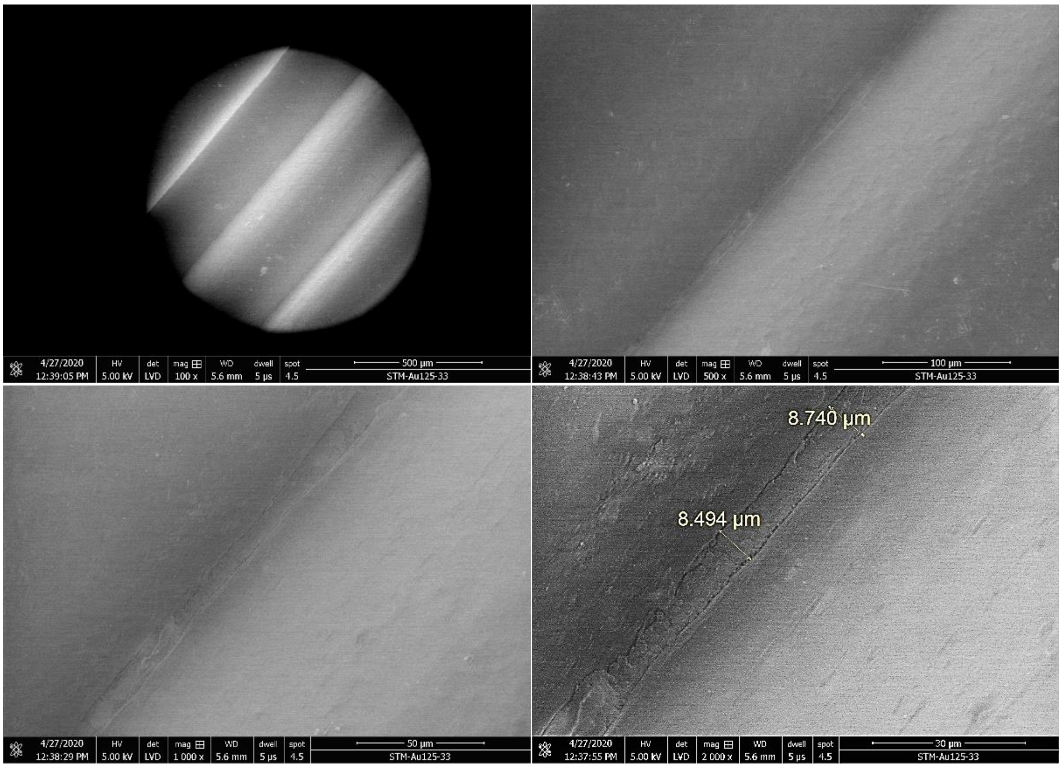

spot 3

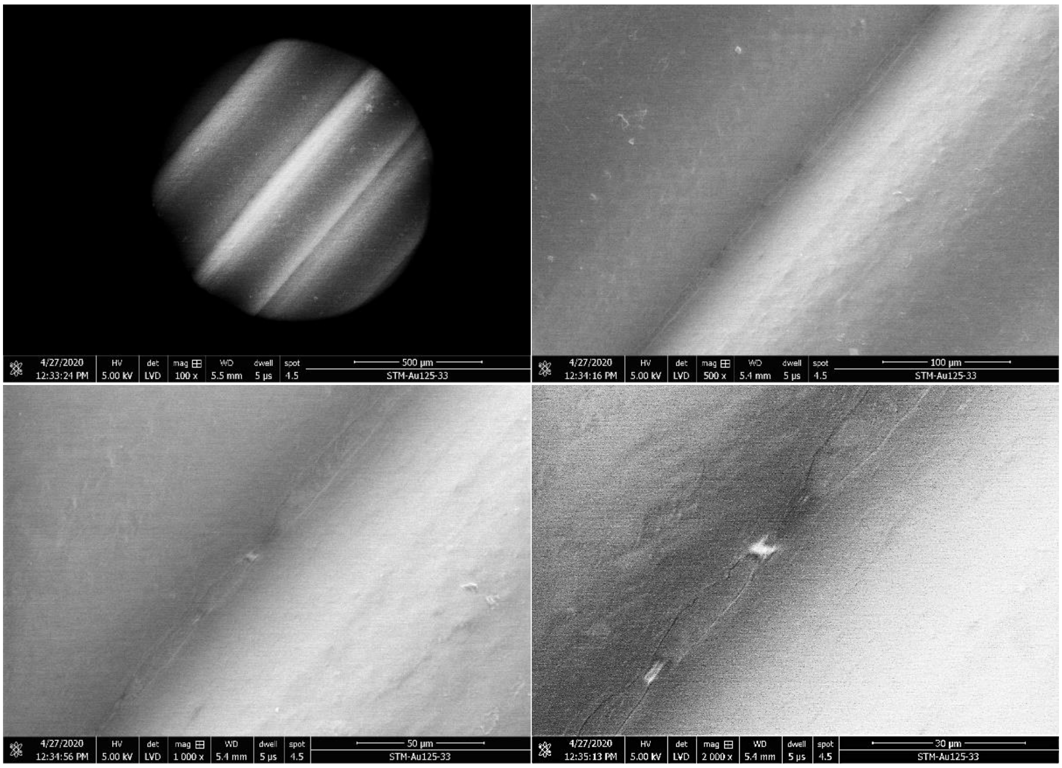
4/27/2020 12:33:24 PM
HV 5.00 kV
det LVD
mag 100 x
WD 5.5 mm
dwell 5 µs
spot 4.5
500 µm
STM-Au125-33
4/27/2020 12:34:16 PM
HV 5.00 kV
det LVD
mag 500 x
WD 5.4 mm
dwell 5 µs
spot 4.5
100 µm
STM-Au125-33
4/27/2020 12:34:56 PM
HV 5.00 kV
det LVD
mag 1 000 x
WD 5.4 mm
dwell 5 µs
spot 4.5
50 µm
STM-Au125-33
4/27/2020 12:35:13 PM
HV 5.00 kV
det LVD
mag 2 000 x
WD 5.4 mm
dwell 5 µs
spot 4.5
30 µm
STM-Au125-33

ethanol 24 h

spot 1

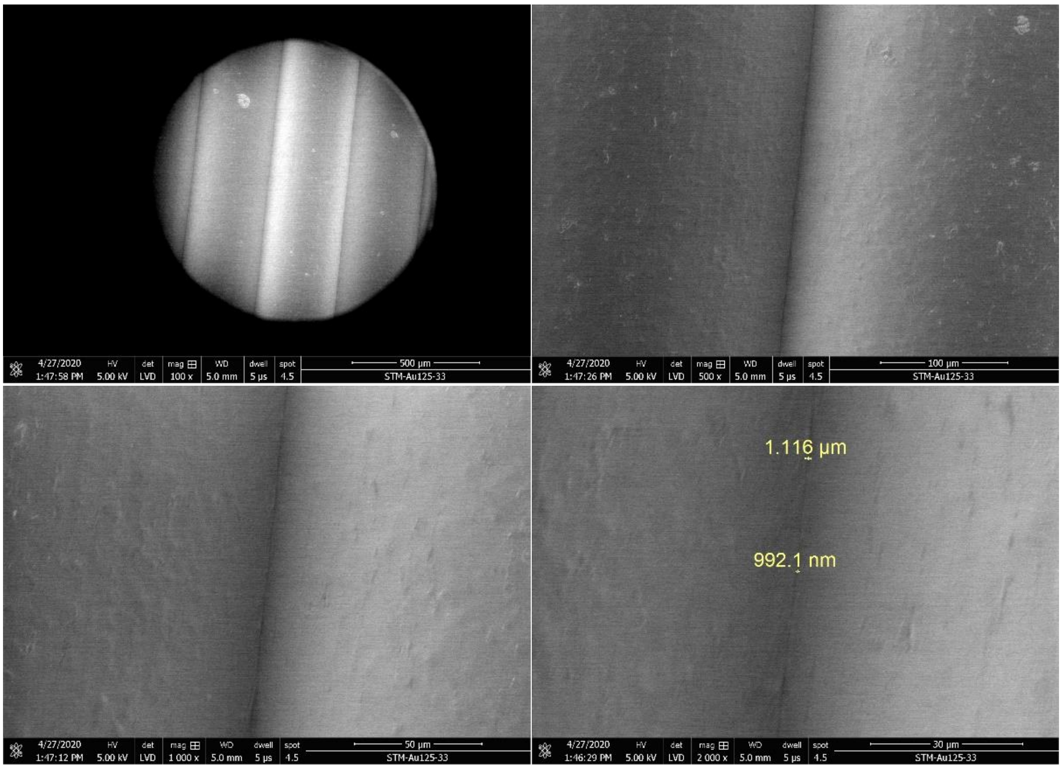

spot 2

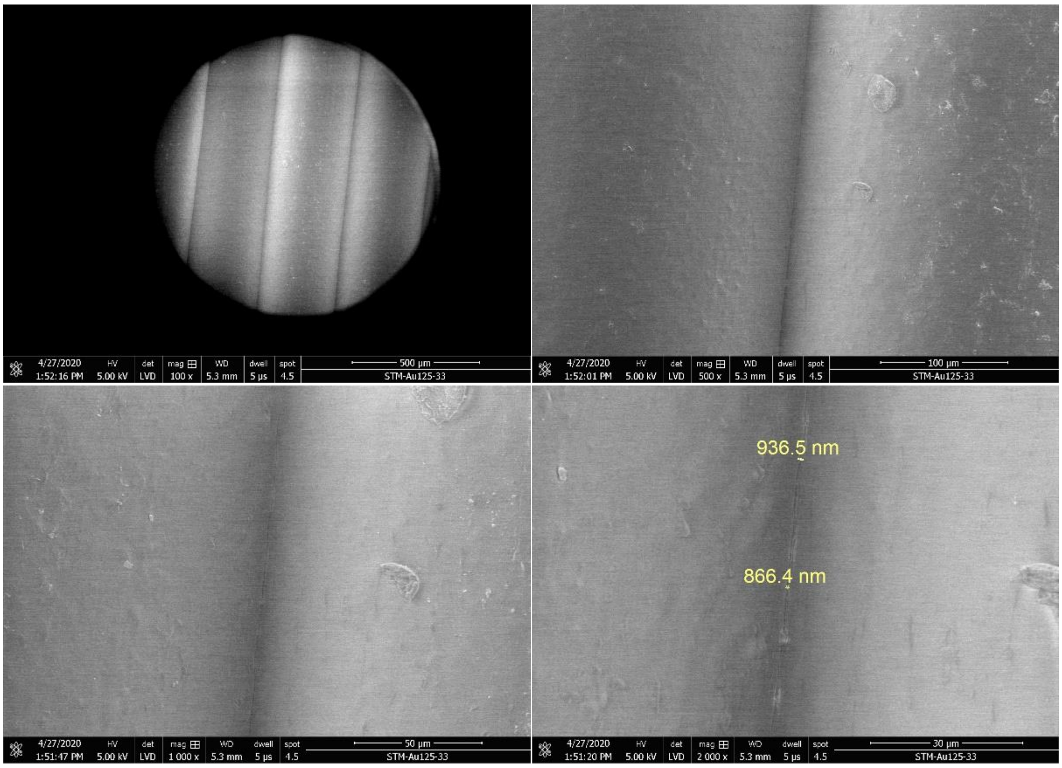

spot 3

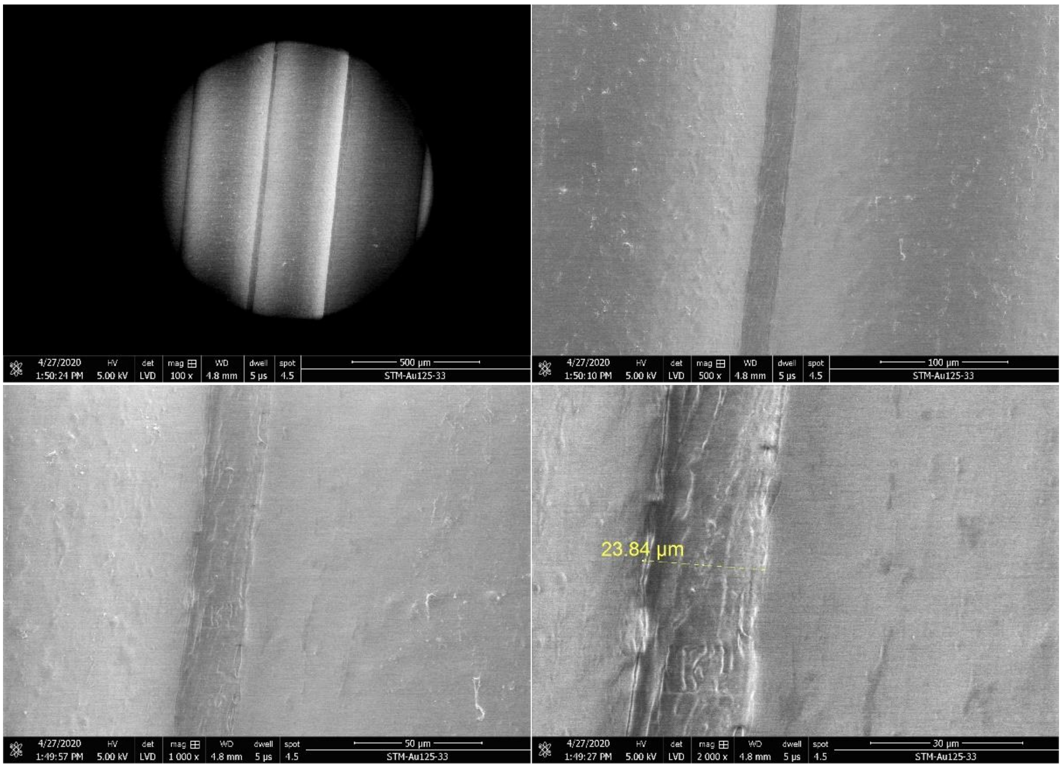
4/27/2020 HV det mag WD dwell spot 500 μm
1:50:24 PM 5.00 kV LVD 100 x 4.8 mm 5 μs 4.5 STM-Au125-33
4/27/2020 HV det mag WD dwell spot 100 μm
1:50:10 PM 5.00 kV LVD 500 x 4.8 mm 5 μs 4.5 STM-Au125-33
4/27/2020 HV det mag WD dwell spot 50 μm
1:49:57 PM 5.00 kV LVD 1 000 x 4.8 mm 5 μs 4.5 STM-Au125-33
23.84 μm
4/27/2020 HV det mag WD dwell spot 30 μm
1:49:27 PM 5.00 kV LVD 2 000 x 4.8 mm 5 μs 4.5 STM-Au125-33

isopropanol 24 h

spot 1

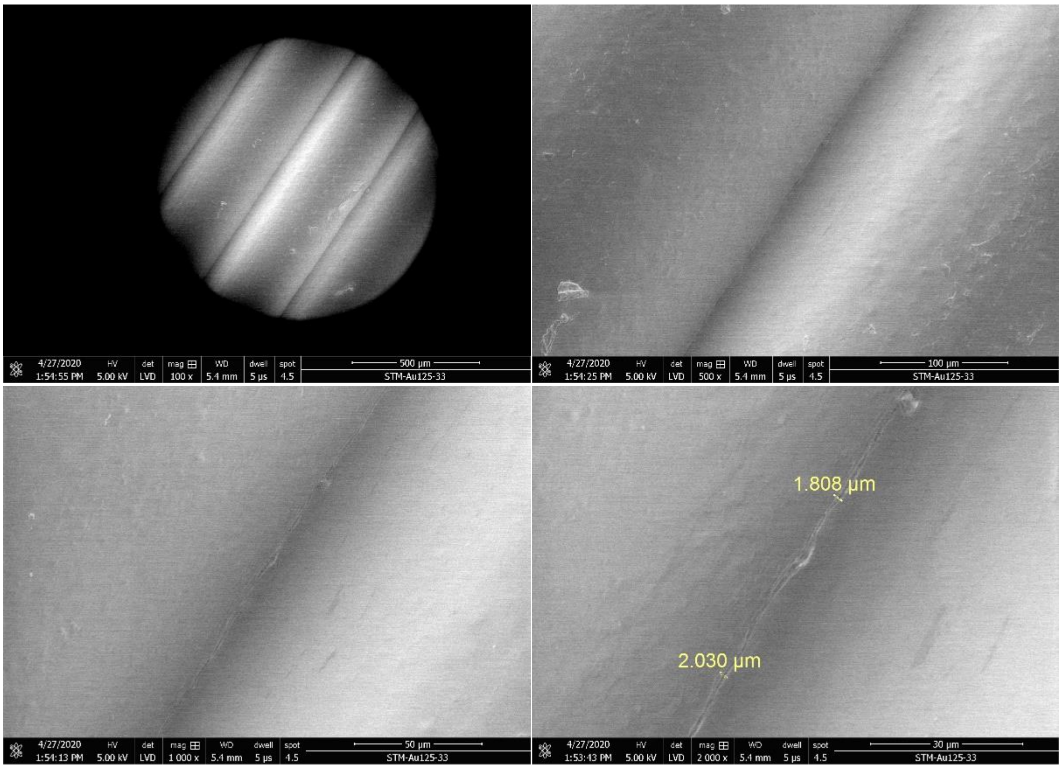

spot 2

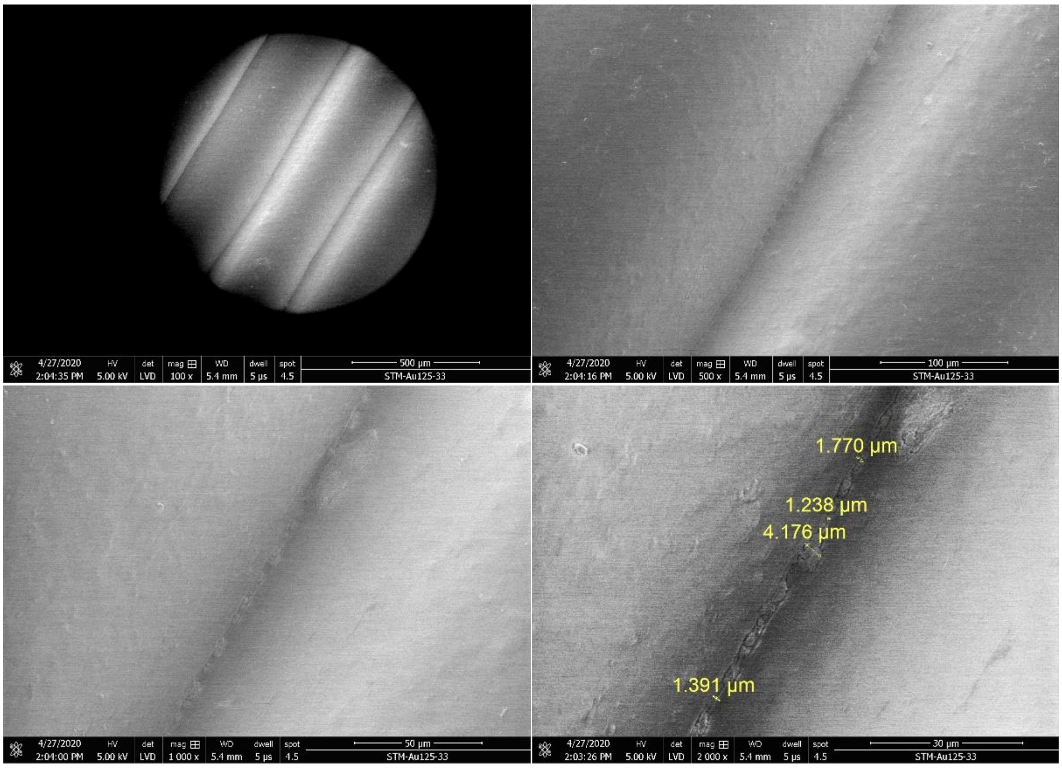

spot 3

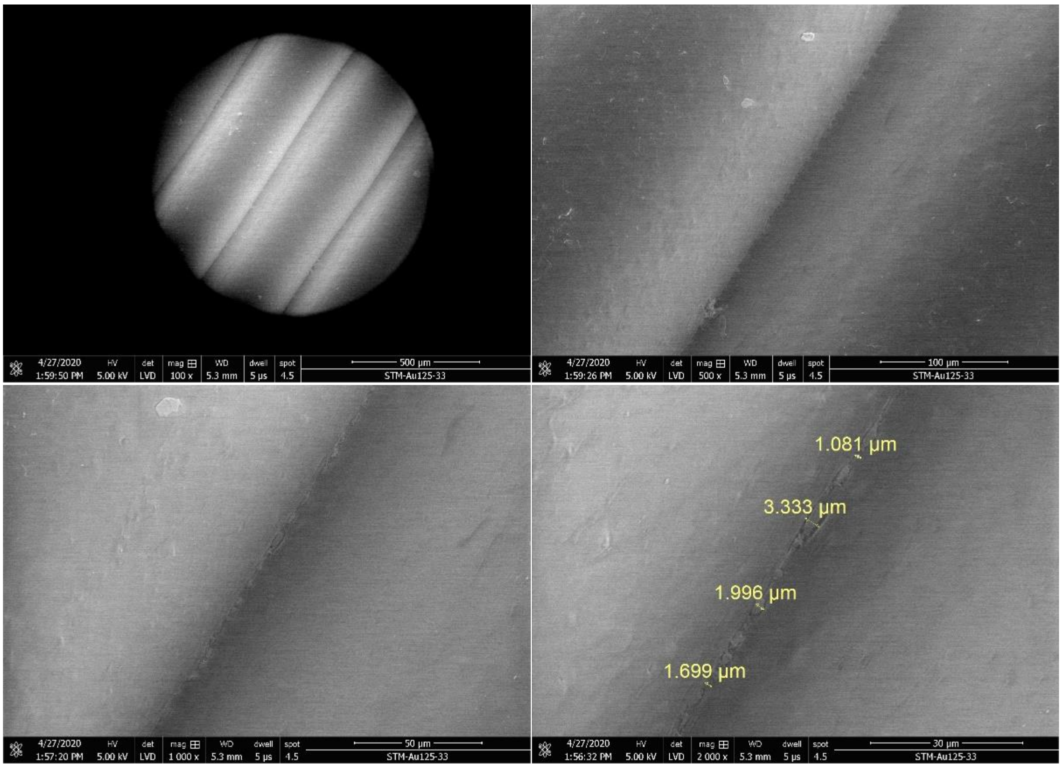

4/27/2020 1:59:50 PM HV 5.00 kV det LVD mag 100 x WD 5.3 mm dwell 5 µs spot 4.5
500 µm
STM-Au125-33
4/27/2020 1:59:26 PM HV 5.00 kV det LVD mag 500 x WD 5.3 mm dwell 5 µs spot 4.5
100 µm
STM-Au125-33
4/27/2020 1:57:20 PM HV 5.00 kV det LVD mag 1 000 x WD 5.3 mm dwell 5 µs spot 4.5
50 µm
STM-Au125-33
1.081 µm
3.333 µm
1.996 µm
1.699 µm
4/27/2020 1:56:32 PM HV 5.00 kV det LVD mag 2 000 x WD 5.3 mm dwell 5 µs spot 4.5
30 µm
STM-Au125-33

sodium hypochlorite 24 h

spot 1

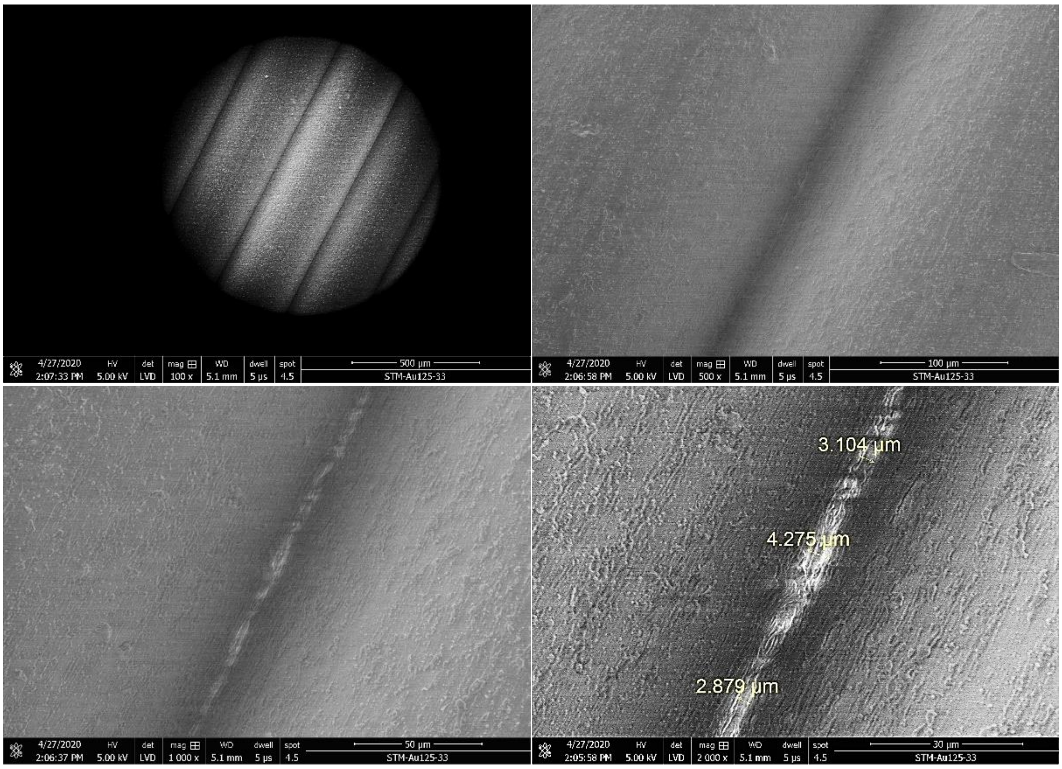

spot 2

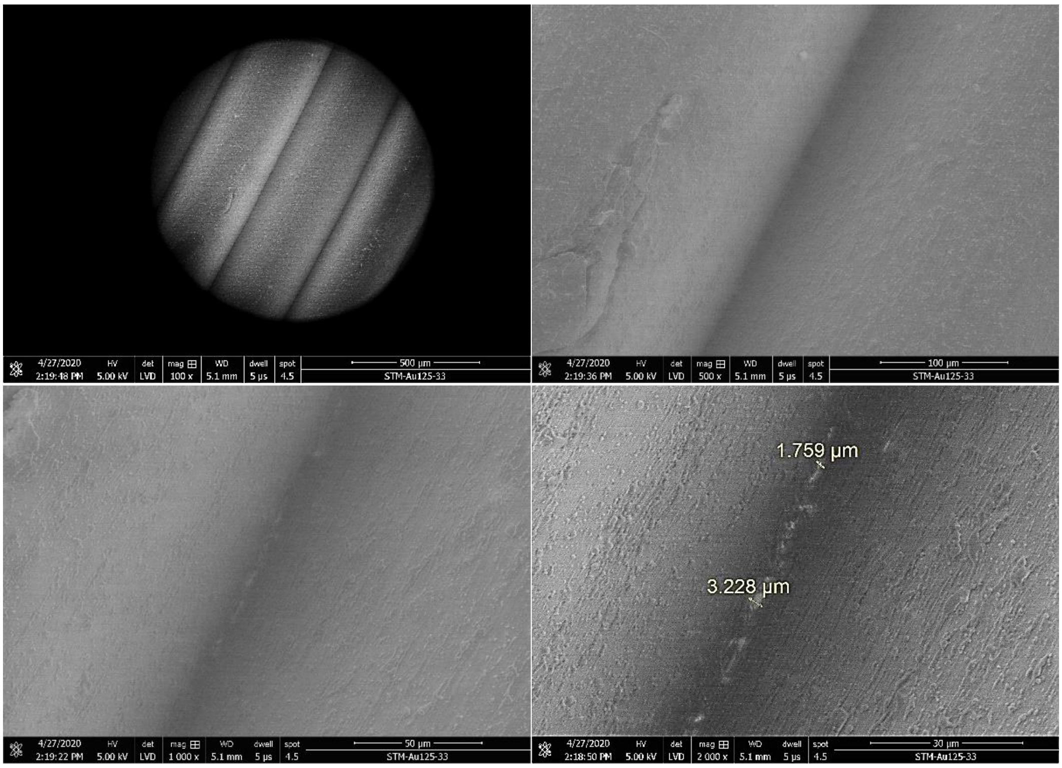

spot 3

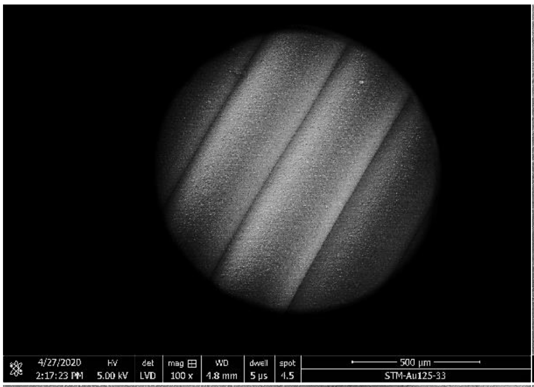
4/27/2020 2:17:23 PM
HV 5.00 kV
det LVD
mag 100 x
WD 4.8 mm
dwell 5 µs
spot 4.5
500 µm
STM-Au125-33

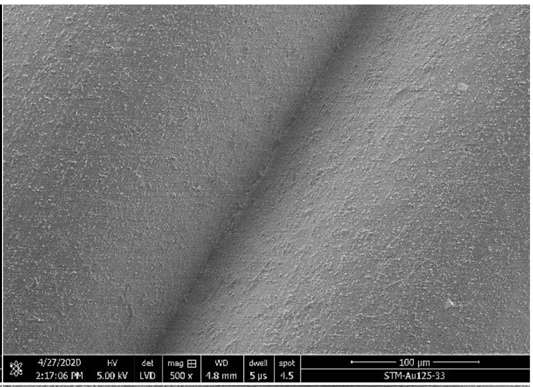
4/27/2020 2:17:06 PM
HV 5.00 kV
det LVD
mag 500 x
WD 4.8 mm
dwell 5 µs
spot 4.5
100 µm
STM-Au125-33

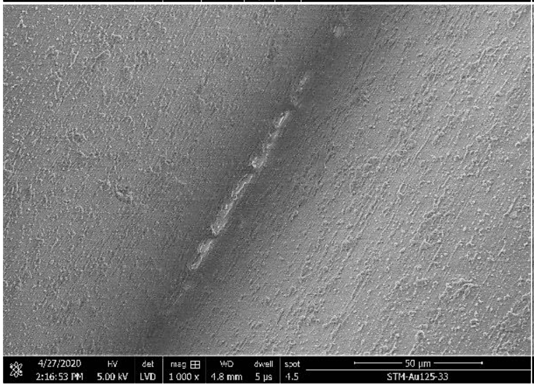
4/27/2020 2:16:53 PM
HV 5.00 kV
det LVD
mag 1 000 x
WD 4.8 mm
dwell 5 µs
spot 4.5
50 µm
STM-Au125-33

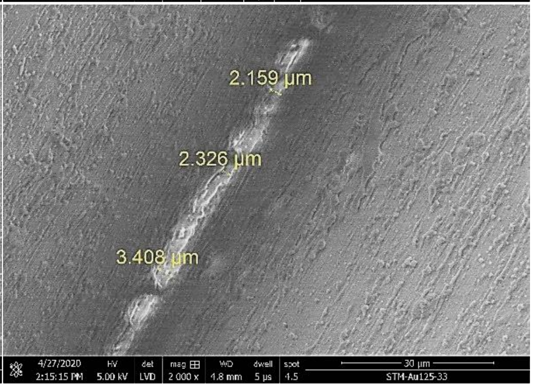
2.159 µm
2.326 µm
3.408 µm
4/27/2020 2:15:15 PM
HV 5.00 kV
det LVD
mag 2 000 x
WD 4.8 mm
dwell 5 µs
spot 4.5
30 µm
STM-Au125-33
